# Sustained response of three pediatric BRAF^V600E^ mutated high-grade gliomas to combined BRAF and MEK inhibitor therapy

**DOI:** 10.18632/oncotarget.26560

**Published:** 2019-01-11

**Authors:** Stephanie A. Toll, Hung N. Tran, Jennifer Cotter, Alexander R. Judkins, Benita Tamrazi, Jaclyn A. Biegel, Girish Dhall, Nathan J. Robison, Kaaren Waters, Palak Patel, Robert Cooper, Ashley S. Margol

**Affiliations:** ^1^ Division of Hematology, Oncology and Blood and Marrow Transplantation, Children's Center for Cancer and Blood Diseases, Children's Hospital Los Angeles, Los Angeles, CA, USA; ^2^ University of Southern California Keck School of Medicine, Los Angeles, CA, USA; ^3^ Kaiser Permanente Southern California, Los Angeles, CA, USA; ^4^ Department of Pathology and Laboratory Medicine, Children's Hospital Los Angeles, Los Angeles, CA, USA; ^5^ Department of Radiology and Imaging, Children's Hospital Los Angeles, Los Angeles, CA, USA

**Keywords:** high-grade glioma, BRAF mutation, targeted therapy, pediatrics

## Abstract

Outcomes for children with high-grade gliomas (HGG) remain dismal despite aggressive treatment strategies. The use of targeted therapy for BRAF^V600E^ mutated malignancies including HGG is being explored as a potentially well tolerated and effective therapeutic option. The results of adult melanoma studies demonstrating that combination therapy with BRAF inhibitors and MEK inhibitors results in prolonged survival led us to employ this treatment strategy in children with BRAF^V600E^ mutated HGG. In this case series, we describe three pediatric patients with HGG with confirmed BRAF^V600E^ mutation who demonstrated responses to combination therapy with dabrafenib and trametinib.

## INTRODUCTION

Astrocytoma is the most common brain tumor in children, with high-grade gliomas (HGG) comprising 11% of all pediatric brain tumors [1]. Survival rates for children with HGG are poor, with 3-year overall survival (OS) of 22 ± 5% despite multi-modal therapy highlighting the need for novel therapeutic options [2].

Alterations in the MAP kinase (MAPK) pathway have been implicated in a number of adult and pediatric malignancies [3–5]. The BRAF^V600E^ point mutation, an amino acid substitution from valine to glutamic acid at position 600 in BRAF, results in constitutive activation of downstream MEK/ERK. This mutation is commonly detected in malignant melanoma and in 10–15% of HGG [5–8].

Clinical trials exploring the use BRAF inhibitors (vemurafenib and dabrafenib) in adults with BRAF^V600E^ mutated malignancies have provided valuable information regarding the efficacy and potential side effects of these agents. Large clinical trials of BRAF inhibitors in adults with BRAF^V600E^ mutated melanoma resulted in tumor response and prolonged progression free survival (PFS) and OS [9, 10]. Unfortunately, most tumors, including those that initially demonstrated significant response, eventually developed resistance resulting in disease progression after six to eight months [9, 10]. Resistance of BRAF mutated malignancies to BRAF inhibitors is often due to reactivation of the MAPK pathway [11–13]. In an attempt to overcome this mechanism of resistance, researchers have combined BRAF inhibitors with downstream MEK inhibitors (trametinib) [12–14]. Combination therapy in adults with BRAF mutated melanoma has yielded higher objective response rates, prolonged survival rates, decreased rates of resistance, and decreased incidence of side effects most notably skin toxicities when compared to BRAF inhibitor monotherapy [9, 10, 15].

Clinical trials examining the use of BRAF inhibitors alone and in combination with MEK inhibitors in children with BRAF^V600E^ mutated malignancies are currently underway. To date, the literature is limited to case reports and series, revealing promising results ranging from partial to complete and sustained responses [16–20]. There are currently no published reports of children with BRAF^V600E^ mutated malignancies treated upfront with BRAF and MEK inhibitor combination therapy.

In this series, we describe three pediatric patients with BRAF^V600E^ mutated HGG treated with dabrafenib and trametinib. Mutational status was assessed using the OncoScan microarray platform (ThermoFisher), which identifies genome-wide copy number and loss of heterozygosity detection as well as a limited number of frequently tested somatic mutations [21]. One patient received the combination as maintenance therapy, one at the time of disease progression, while the third patient was treated with combination therapy at initial diagnosis.

## PATIENT 1

**Figure 1:**
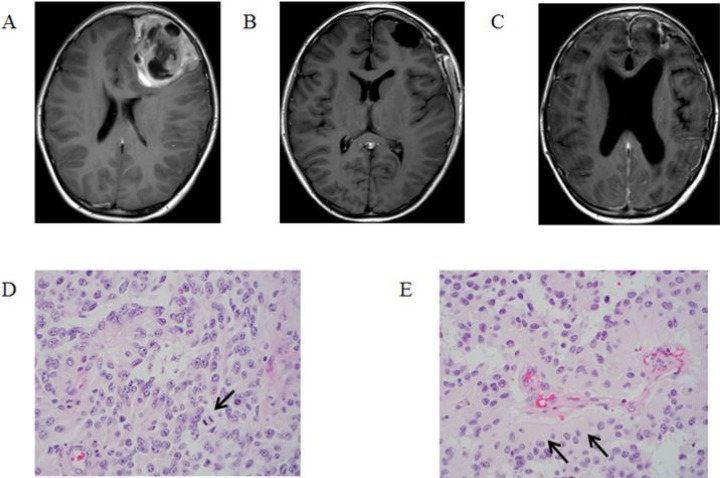
T1-Weighted MRI post gadolinium-based contrast images: (**A**) at diagnosis, (**B**) post resection, and (**C**) at time of recurrence. Histopathologic review of tumor specimen after resection: (**D**) a moderately to highly cellular neoplasm with scattered mitotic figures (arrow) and a pseudopapillary growth pattern, and (**E**) pseudorosettes with thick cytoplasmic processes extending towards the vessels (arrows).

A 13-year-old male presented with a one-month history of fatigue, emesis and diffuse headache. Computed tomography (CT) and subsequent magnetic resonance imaging (MRI) revealed a 5.9 × 6.5 × 6.4 cm cortically based, heterogeneously enhancing mass involving the left frontal lobe (Figure 1A). He underwent a gross total resection of the tumor (Figure 1B) and pathology was consistent with anaplastic astroblastoma (Figure 1D, 1E). Molecular testing using the OncoScan microarray platform revealed a tetraploid tumor with four copies of all the autosomes, except for chromosome 1, and two copies of each X and Y. Analysis revealed a BRAF^V600E^ mutation and a copy number loss of chromosome 9 encompassing CDKN2A/B.

Following resection, the patient underwent focal radiation therapy (59.4 Gy in 33 fractions) with concurrent temozolomide (90 mg/m^2^/day). Post-irradiation, the patient was started on maintenance therapy with dabrafenib (4.5 mg/kg/day divided twice daily) and trametinib (2 mg/day once daily). One month after starting maintenance therapy, he developed mild fatigue. Trametinib was discontinued six months later due to family preference. He had no other treatment-attributable toxicities. The patient remained disease free for 20 months at which time he presented with disseminated disease recurrence and died 2 months later (Figure 1C).

## PATIENT 2

**Figure 2:**
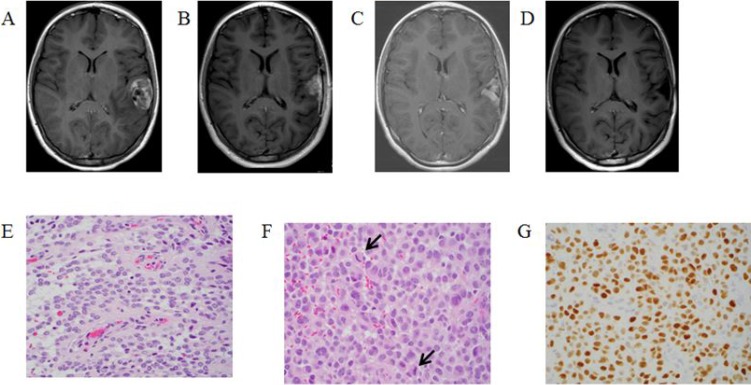
T1-Weighted MRI post gadolinium-based contrast images: (**A**) at diagnosis, (**B**) one month post initial resection demonstrating recurrent disease, (**C**) after third resection and irradiation demonstrating tumor progression (**D**) after two months of targeted therapy demonstrating tumor response. Histopathologic review of tumor specimen (**E**) after second resection showing both solid and perivascular growth pattern, (**F**) after third resection more pronounced epithelioid morphology, moderate nuclear pleomorphism, and increased mitotic activity (arrows), (**G**) after third resection showing nuclear olig2 expression was strong in tumor cells, consistent with a glioma.

A 12-year-old female presented with a three-week history of diffuse headache and three days of diplopia and blurry vision. Initial head CT demonstrated edema in the left temporal and frontal lobes. Subsequent MRI revealed a 3.8 × 2.4 × 3.1 cm cortically based mass within the left superior temporal gyrus (Figure 2A).

The patient underwent a gross total resection of the lesion and histopathology was most consistent with an ependymoma. The initial plan was close observation, and MRI performed one month post-operatively demonstrated new infiltrating tumor within the resection cavity (Figure 2B). The patient underwent a partial re-resection (Figure 2E) followed by two cycles of chemotherapy (cisplatin, cyclophosphamide, etoposide, and vincristine). Post-chemotherapy MRI again demonstrated tumor progression requiring additional surgery. Histopathology and immunohistochemistry analysis at the time of the third resection (Figure 2F and 2G) were more consistent with HGG and OncoScan revealed a BRAF^V600E^ mutation. OncoScan also detected numerous copy number abnormalities including homozygous copy number loss at chromosome 9 involving the CDKN2A/B locus. The patient underwent focal radiation therapy (54 Gy in 30 fractions) with concurrent temozolomide. MRI obtained one-month post chemoradiotherapy again showed tumor progression (Figure 2C). At that time the patient was started on BRAF inhibitor monotherapy (dabrafenib 4.5 mg/kg/day divided twice daily). MRI performed two months later demonstrated a significant decrease in tumor size (Figure 2D). Six months into treatment with dabrafenib, trametinib was added (2 mg/day). She has had no dose-modifying toxicities. The patient has remained on therapy with a small amount of stable disease for 32 months.

## PATIENT 3

**Figure 3:**
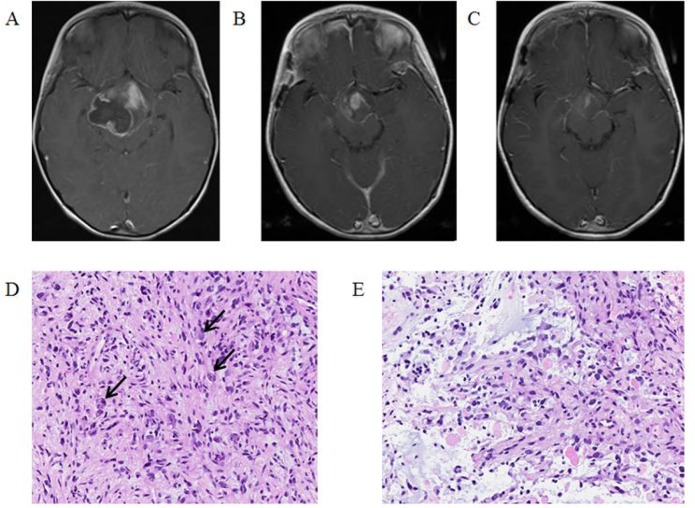
T1-Weighted MRI post gadolinium-based contrast images: (**A**) at diagnosis, (**B**) after three months of targeted therapy showing 85% decrease in tumor size, (**C**) after eight months of targeted therapy showing a further decrease in tumor size. Histopathologic review of tumor after resection: (**D**) a moderately cellular tumor with large polygonal ganglion cells (arrows) among smaller, elongated astrocytic cells, (**E**) frequent mitotic figures and numerous eosinophilic granular bodies.

A four-year-old female presented after an episode of headache followed by loss of consciousness. On physical exam she was noted to have a left sided visual field deficit. MRI of the brain demonstrated a 6 × 4.6 × 5 cm mass centered in the hypothalamus with expansion into the suprasellar area and pons with intratumoral hemorrhage (Figure 3A). The patient underwent a biopsy and ventriculoperitoneal shunt placement. Pathology was consistent with anaplastic ganglioglioma (Figure 3D, 3E) and OncoScan revealed a BRAF^V600E^ mutation. In addition to the BRAF^V600E^ mutation, a deletion on the short arm of chromosome 4 and numerous copy number alterations spanning chromosome 22 were also discovered. No CDKN2A loss was identified. Given the young age of the child and desire to avoid irradiation, the decision was made to proceed with targeted therapy. The patient was started on dabrafenib (4.5 mg/kg/day divided twice daily) upfront with the addition of trametinib (0.025 mg/kg/day) one month later. Four weeks after initiation of therapy her visual deficit resolved. MRI obtained three months after initiation of therapy demonstrated an 85% decrease in tumor size (Figure 3B). MRI performed after eight months of therapy demonstrated a further decrease in size (Figure 3C).

The patient reports no side effects and has remained on therapy with stable disease for 23 months.

Dermatologic exams and echocardiograms were conducted every three months and ophthalmologic evaluations were completed every three to six months for all patients. None of the patients developed any significant dermatologic, cardiac or ophthalmologic findings.

## DISCUSSION

Despite efforts over the past several decades, survival rates for children with HGG remain dismal [2]. While extent of resection is a major factor in survival, it puts patients at risk for devastating neurologic deficits. Adjuvant therapy is associated with many short and long term effects and offers little survival benefit [22, 23]. Given the lack of effective therapies for patients with HGG, the use of molecularly targeted agents is of particular interest in the treatment of this disease.

BRAF inhibitor plus MEK inhibitor combination therapy has proved to be effective in the treatment of BRAF mutated malignancies. Investigations of combination therapy in murine models of BRAF^V600E^ mutated HGG have demonstrated sustained MAPK pathway inhibition, prolonged survival and decreased cutaneous toxicity when compared to monotherapy [24, 25]. Adults with BRAF mutated melanoma have higher responses rates and prolonged PFS and OS when treated with combination therapy compared to BRAF inhibitor monotherapy [15, 26, 27]. Flaherty *et al.* reported an overall response rate (ORR) of 76% in the combination therapy group (dabrafenib and trametinib) compared to 54% in those receiving monotherapy (*P* = 0.03) while Long *et al.* reported an ORR of 67% in subjects receiving combination therapy compared to 51% in those receiving dabrafenib alone (*P* = 0.002) [15]. Additionally, in a study comparing the combination of dabrafenib and trametinib with vermurafenib monotherapy, Robert *et al.* reported an ORR of 64% in the combination group and 51% in the vemurafenib group (*P* < 0.001) [27].

The efficacy of BRAF inhibitors alone in the treatment of children with recurrent/progressive BRAF^V600E^ mutated HGG has been reported [16–20]. Robinson *et al.* reported a child with a recurrent BRAF^V600E^ mutated glioblastoma multiforme who achieved a complete response after four months of treatment with vemurafenib [16]. A study of dabrafenib monotherapy in children with recurrent/progressive solid tumors has reported the outcomes of eight subjects with HGG: three complete responses, three partial responses and two with disease progression [17]. To date there is one case report of a child treated with combination therapy. Marks, *et al.* reported a patient with recurrent BRAF^V600E^ mutated anaplastic ganglioglioma who demonstrated a complete response to treatment with dabrafenib and trametinib [20].

While BRAF inhibitors have proven effective, their ultimate success may be limited in some patients by the development of resistance. Many mechanisms of resistance have been described, several of which are due to increased RAF dimerization. While BRAF inhibitors sufficiently block signaling by BRAF monomers that are present in BRAF^V600E^ mutated cells, they induce RAF dimerization in BRAF wild-type cells [28, 29]. Splice variants of BRAF also demonstrate increased dimerization in the presence of BRAF inhibitors [11]. The ultimate effect of increased dimerization is paradoxical activation of MAPK pathway. Given that reactivation of MAPK pathway activation is a major mechanism of resistance, combination therapy to block downstream MEK is an effective treatment strategy for many patients.

The use of dabrafenib in combination with trametinib in children with newly diagnosed BRAF^V600^ mutated LGG and relapsed/refractory HGG is currently being investigated (https://clinicaltrials.gov: NCT02684058). Additionally, there are clinical trials under development and in progress examining the use of second generation BRAF inhibitors in children and adults with BRAF mutated malignancies (https://clinicaltrials.gov: NCT03429803, NCT02428712).

However, for the use of BRAF inhibitor monotherapy still raises the concern for development of serious side effects, particularly cutaneous side effects such as rash and squamous cell carcinoma. Studies of the use of combination therapy in adult BRAF mutated melanoma have demonstrated a decreased incidence of cutaneous side effects when compared to BRAF inhibitor monotherapy. Robert *et al.* reported that cutaneous adverse events were more common in subjects receiving vemurafenib versus those receiving combination therapy including rash (43% vs. 22%), photosensitivity reaction (22% vs. 4%), skin papillomas (23% vs. 2%) and squamous-cell carcinomas and keratoacanthomas (18% vs. 1%) [27]. The pediatric patient described by Marks, *et al.* experienced a morbilliform rash consistent with a Type IV allergic reaction when treated with vemurafenib. When the patient was later treated with a combination of dabrafenib and trametinib, the patient experienced no cutaneous side effects [20].

Here we describe the safe and effective use of combination BRAF and MEK inhibitor therapy in three children with BRAF^V600E^ mutated HGG. We also describe for the first time the use of combination BRAF and MEK inhibitor therapy as an upfront treatment strategy for a pediatric patient. Importantly, all patients tolerated the combination of BRAF and MEK inhibitor therapy well with minimal side effects.

In conclusion, this case series presents evidence that combination BRAF and MEK inhibitor therapy is an effective and appropriate treatment option and that it is safe for children with BRAF^V600E^ mutated HGG. And although we cannot draw conclusions based on this series, our experience highlights the importance of molecular testing and supports the ongoing development of clinical trials examining the use of combination therapy for children with BRAF^V600E^ mutated HGG including those with newly diagnosed disease.
