# Addendum: Sustained response of three pediatric BRAF^V600E^ mutated high-grade gliomas to combined BRAF and MEK inhibitor therapy

**DOI:** 10.18632/oncotarget.28445

**Published:** 2023-05-19

**Authors:** Stephanie A. Toll, Hung N. Tran, Jennifer Cotter, Alexander R. Judkins, Benita Tamrazi, Jaclyn A. Biegel, Girish Dhall, Nathan J. Robison, Kaaren Waters, Palak Patel, Robert Cooper, Ashley S. Margol

**Affiliations:** ^1^Division of Hematology, Oncology and Blood and Marrow Transplantation, Children’s Center for Cancer and Blood Diseases, Children’s Hospital Los Angeles, Los Angeles, CA, USA; ^2^University of Southern California Keck School of Medicine, Los Angeles, CA, USA; ^3^Kaiser Permanente Southern California, Los Angeles, CA, USA; ^4^Department of Pathology and Laboratory Medicine, Children’s Hospital Los Angeles, Los Angeles, CA, USA; ^5^Department of Radiology and Imaging, Children’s Hospital Los Angeles, Los Angeles, CA, USA; ^*^These authors have contributed equally to this work

**This article has an addendum:** Signed HIPPA forms from the parents of the 2 patients who were alive at the time of manuscript submission were obtained.

Original article: Oncotarget. 2019; 10:551–557. 551-557. https://doi.org/10.18632/oncotarget.26560

